# Accumulation and Release of Mercury in the Lichen *Evernia prunastri* (L.) Ach

**DOI:** 10.3390/biology10111198

**Published:** 2021-11-18

**Authors:** Andrea Vannini, Muhammad Bilal Jamal, Margherita Gramigni, Riccardo Fedeli, Stefania Ancora, Fabrizio Monaci, Stefano Loppi

**Affiliations:** 1 Department of Life Sciences, University of Siena, 53100 Siena, Italy; andrea.vannini@unisi.it (A.V.); muhammad.jamal@student.unisi.it (M.B.J.); margherita.gramig@student.unisi.it (M.G.); riccardo.fedeli@student.unisi.it (R.F.); fabrizio.monaci@unisi.it (F.M.); 2 Department of Physics, Earth and Environmental Sciences, University of Siena, 53100 Siena, Italy; stefania.ancora@unisi.it; 3 BAT Center—Interuniversity Center for Studies on Bioinspired Agro-Environmental Technology, University of Naples ‘Federico II’, 80138 Napoli, Italy

**Keywords:** bioaccumulation, biomonitoring, Hg, photosynthesis, toxicity, uptake

## Abstract

**Simple Summary:**

Lichens are among the most used and most effective biomonitors of airborne mercury (Hg); however, although the ability of lichens to take up Hg and provide accurate patterns of Hg contamination around emission sources is well documented, information on their ability to reflect the decreasing environmental availability of this element is minimal and contrasting. The aim of this study was to investigate both the accumulation and release of Hg^2+^ in lichens, using *Evernia prunastri* as a model species, and hypothesizing that 24 months is sufficient for treated samples to return to background values. The results of this study highlighted the ability of the lichen *E. prunastri* to reflect very quickly the available Hg concentration, as well as to indicate an ameliorated situation (e.g., the closure of an Hg source). However, we have found evidence that an acute pollution episode can influence the content of Hg in lichens for several years.

**Abstract:**

This study investigated the dynamics of the accumulation and release of Hg^2+^ in lichens, using *Evernia prunastri* (L.) Ach. as a model species. Thalli were incubated with solutions containing 1, 10, and 100 µM Hg^2+^ and then exposed for 1, 2, 3, 6, 12, 18, and 24 months at the Botanical Garden of the University of Siena (a location free from local Hg sources). Lichen samples accumulated Hg proportionally to the exposure concentration, and after the exposure, reductions over time were evident, already starting from 1–2 months. After 24 months, samples released 72–74 (healthy thalli) to 94% (unhealthy thalli) of the accumulated Hg, but control values of untreated samples were never reached. Depending on the Hg content after the exposure, stable decreased concentrations were reached after 6–24 months. The results of this study highlight the ability of the lichen *E. prunastri* to reflect rapidly increasing environmental Hg concentrations, as well as to indicate an ameliorated situation (e.g., the closure of an Hg source). However, we have found evidence that an acute pollution episode can influence the content of Hg in lichens for several years.

## 1. Introduction

The release of potentially toxic elements (PTEs) in the environment is of great concern for human health, as they may accumulate in the body and give rise to a wide array of toxicological effects [1]. Mercury (Hg) is probably the PTE of greatest concern, being neurotoxic (i.e., methylmercury, MeHg) and undergoing long-range atmospheric transport, mostly in the elemental form [2,3]. Although Hg can be emitted from natural sources such as volcanic eruptions, human activities, such as mining and fossil fuel combustion, have led to widespread global Hg pollution. Measures for limiting Hg emissions into the environment have been put into action over the years [4,5], but despite this, the global emissions of Hg are still increasing by 1.8% [6].

Mercury is highly mobile in the atmosphere, and even small releases of this element into the environment can result in a significant exposure [3]. As a consequence, it is extremely important to monitor the fate of Hg emissions [7]. Methods for sampling and analysis of atmospheric mercury species are demanding and expensive [8]. Biological monitoring is thus of paramount importance for assessing the presence of Hg in the environment [9], and lichens are among the most used and most effective biomonitors of airborne Hg [10]. Thanks to their strict dependence on the atmosphere for mineral nutrition and the lack of protective structures, such as cuticle and stomata in the leaves of vascular plants, lichens can take up Hg proportionally to its environmental concentration [11,12]. Lichens are known to accumulate both elemental (Hg^0^) and ionic (Hg^2+^) mercury, i.e., the two main forms of atmospheric Hg [13], up to very high concentrations, even 4–5 orders of magnitude higher than those found in samples grown in remote areas [14,15], without showing signs of physiological stress, despite the known phytotoxicity of this element [16]. Mercury is accumulated mainly in the elemental and ionic forms [14,15], but in some peculiar cases, such as highly contaminated mining areas, the exposition of particulate Hg may also relevantly contribute to the total Hg content of lichen samples [17,18].

Although the ability of lichens to take up Hg and provide accurate patterns of Hg contamination around emission sources is well documented [11,19,20,21,22,23,24], information on their ability to reflect the decreasing environmental availability of this element is minimal and contrasting. Walther et al. [25] found an efficient long-term ability of lichens to release the accumulated Hg, as a consequence of the phase-out of the contamination source (a chlor-alkali facility), but Godinho et al. [26] and Vannini et al. [15] reported the lack of long- and short-term release of accumulated Hg over time. As a matter of fact, there is the need for experimental studies investigating the ability of lichens to release Hg once accumulated, especially in the case of the ionic form, which is by far the lesser known form.

This study was thus undertaken with the aim of investigating both the accumulation and release of Hg^2+^ in lichens, using *Evernia prunastri* as a model species, and hypothesizing that 24 months is sufficient for treated samples to return to background values.

## 2. Materials and Methods

### 2.1. Sample Collection

*Evernia prunastri* (L.) Ach. is a common epiphytic (tree inhabiting) lichen widely used in biomonitoring and laboratory studies and has a known ability to accumulate Hg proportionally to its environmental availability [15]. Thalli of *E. prunastri* were collected from branches of *Prunus spinosa* shrubs growing in a remote area of Tuscany, in central Italy (43°11′60″ N, 11°21′33″ E, 310 m a.s.l.), located far from any local source of contamination. In the laboratory, thalli were freed of any extraneous material, such as bark residues and other lichen species. Then, the samples were washed with deionized water to remove dust particles simply deposited onto the lichen surface and air-dried for two days.

### 2.2. Experimental Design

The whole pool (ca. 60 g) of washed lichen thalli was randomly divided into four batches of 15 g each, which were incubated for 1 h in solutions containing Hg^2+^ at concentrations of 0 (control), 1, 10, and 100 µM. Treatment solutions were prepared by dissolving mercury chloride (HgCl_2_) in deionized water. The treatment concentrations were selected in order to guarantee a consistent Hg uptake in the thalli [14]. 

**Figure 1:**
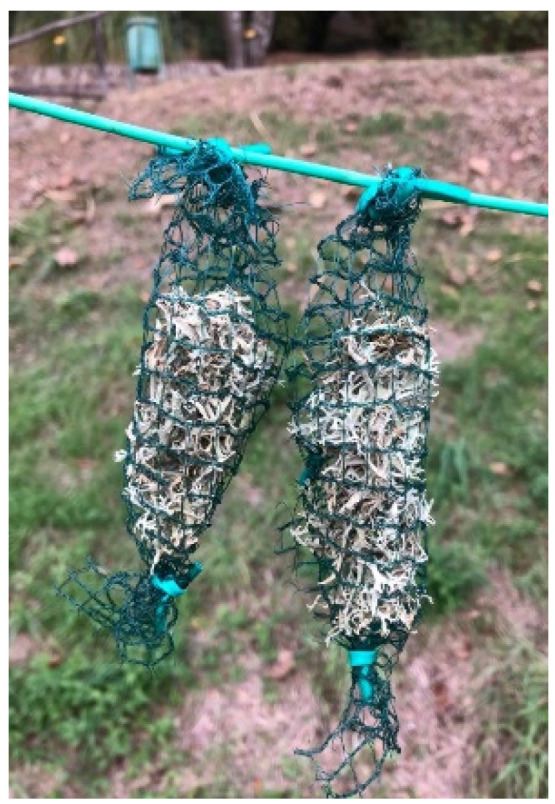
Lichen bags of *Evernia prunastri* exposed in the Botanical Garden of the University of Siena.

After incubation, samples were shaken by hand and the excess water was removed using paper towels. The samples were then air-dried at room temperature and each batch was subdivided into 160 samples of approximately 300 mg, loosely wrapped inside a nylon net (lichen bags [27]). After the treatments, the lichen bags were exposed in the open field (i.e., to the weather conditions) at the Botanical Garden of the University of Siena at a height of about 2 m above the ground, using a nylon thread as support (Figure 1). Prior to the transplantation, from each of the four sample pools, six bags (statistical replicates) were collected, stored at −20 °C, and used later as a starting point for the evaluation of the Hg release (T_0_). The samples were retrieved after 1, 2, 3, 6, 12, 18, and 24 months from the exposure [27]. After removal, all samples were air-dried overnight in a climatic chamber at 16 °C and 55% RH, and then stored in plastic containers at −20 °C until chemical analyses. 

### 2.3. Chemical Analysis

For the chemical analysis of the mercury content in lichen samples, the method suggested by Tretiach et al. [28] was followed. About 400 mg of dried thalli from each lichen bag was weighed in polytetrafluoroethylene (PTFE) closed vessels of a microwave digestion system (Ethos 1, Milestone, Shelton, CT, USA) equipped with a temperature sensor. The samples were digested with 8 mL of concentrated reagent-grade HNO_3_ and 2 mL of 30% (*w*/*v*) H_2_O_2_ by applying to the closed vessel a stepwise power program (250, 0, 250, 400, and 650 W). After cooling, the digested solution was transferred to polyethylene (PE) conical tubes, adjusted to 50 mL using deionized Milli-Q water, and analyzed for total concentrations of Hg using a Flow Injection Mercury System (FIMS 400, Perkin Elmer, Waltham, MA, USA). Instrumental calibration was performed using an aqueous multielement reference solution starting from commercial stock solutions at a concentration of 1000 mg/L, prepared immediately before use. Element concentrations were determined by the method of standard additions and are expressed in µg/g on a dry weight basis. Sample homogeneity and uncertainties related to digestion and analysis were checked by replicate determinations, while accuracy was checked by routine Hg determinations in standard reference materials (SRM no. 1515 “Apple Leaves”, 1573 “Tomato Leaves”, and IAEA-336 “Lichen”) from the National Institute of Standards and Technology (Gaithersburg, MD, USA).

### 2.4. Physiological Parameters

As the photobiont is known to be the target of mercury accumulation [29], and Hg is known to be toxic for the lichen photobiont at exposure concentrations above 50 μM [14], selected photosynthetic parameters, such as the photosynthetic efficiency and the chlorophyll *a* content, were measured in order to assess the viability of the photobiont (i.e., *Trebouxia* algae) after the treatments.

The photosynthetic efficiency, expressed as F_V_/F_M_, where F_V_ indicates the difference between the maximal (F_M_) and the basal (F_0_) fluorescence, was measured using a plant efficiency analyser Handy PEA (Hansatech instruments Ltd., Norfolk, UK). The analyses were carried out by flashing dark-adapted samples (ca. 15 min) with a saturating (1800 μmol/m^2^/s) red light (650 nm) pulse for 1 s. Fifteen measurements were taken from each replicate.

Chlorophyll *a* content was measured by liquid chromatography (Agilent 1100 system). Samples (ca. 50 mg) were homogenized in 1 mL of dimethylformamide (DMF) and then centrifuged at 15,000 rpm for 5 min. The supernatant was filtered at 0.45 µm using a syringe filter and then directly analyzed by HPLC. Chlorophyll *a* separation was granted using an Agilent C18 column (250 × 5 mm; pore size 5 µm) as the stationary phase and methanol and acetone (50:50) as the mobile phase and eluted isocratically at 1 mL/min. Quantification was performed using a calibration curve of pure chlorophyll *a* standard (Merck, Darmstadt, Germany) in the range of 5–100 µg/mL. Runs were monitored at 440 nm. The precision of the analysis was estimated by analyzing the same sample five times, and was always >98%.

### 2.5. Statistical Analysis

Statistically significant differences between Hg concentrations across time were checked by means of the pairwise permutation t-test, applying the Benjamini–Hochberg correction for multiple testing [30]. Release of Hg across time was modeled by applying exponential and power regression analysis; best correlations were evaluated by comparing R^2^ and AIC (Akaike information criterion) values. Release rates (mean µg/g (Hg) month^−1^) were then modeled by deriving the equations previously obtained, as reported by Vannini et al. [27]. To account for possible variations in the expression of physiological parameters over time, these results were expressed as the ratio between treated and control samples. Statistically significant differences between variations in the ratio across time were checked using the Wilcoxon signed-rank test, applying the above-mentioned correction for multiple testing. All calculations were run using the free software R [31].

## 3. Results

**Figure 2:**
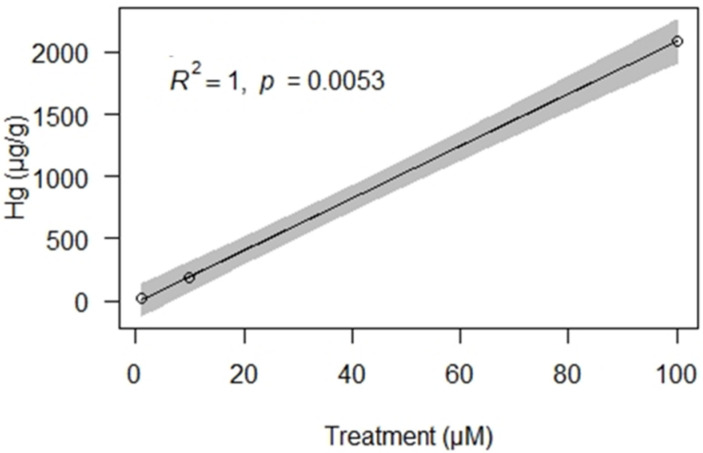
Linear regression between the content of Hg in the treatment solutions and in the lichen *Evernia prunastri*. Gray area = 95% confidence interval.

**Figure 3:**
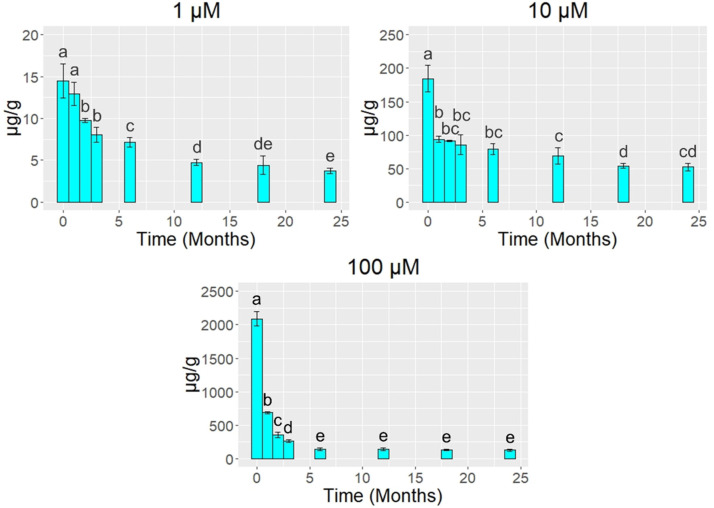
Concentrations of Hg (mean ± standard error) in samples of the lichen *Evernia prunastri* incubated with 1, 10, and 100 µM Hg^2+^ solutions and transplanted for 1, 2, 3, 6, 12, 18, and 24 months at the Botanical Garden of the University of Siena. Different letters indicate statistically significant (*p* < 0.05) differences between concentrations over time.

The lichen *E. prunastri* accumulated Hg^2+^ proportionally to the concentration in the treatment solution (Figure 2). After the exposure at the Botanical Garden, all samples showed Hg reductions across time, already starting from 1–2 months (Figure 3). More in detail, samples treated with the lowest Hg concentration (1 µM) showed a statistically significant decrease after two months (−33%; *p* < 0.05), with reductions of 51% after 6 months, 70% after 18 months, and 74% after 24 months; significant differences were not found after 18 months (*p* > 0.05). Samples treated with the medium and highest Hg concentration (10 and 100 µM, respectively) showed a statistically significant decrease already after one month (*p* < 0.05). In detail, samples treated with 10 µM Hg showed releases of 49% after one month, 57% after 6 months, 71% after 18 months, and 72% after 24 months; significant differences were not found after 18 months (*p* > 0.05). Samples treated with 100 µM Hg showed decreases of 67% after one month, 93% after 6 months, and 94% after 18 and 24 months; significant differences were not found after 18 months (*p* > 0.05). 

At the end of the experiment, treated samples showed Hg concentrations 16, 228, and 550 (1, 10, and 100 µM, respectively) times higher than control samples (0.23 ± 0.09, µg/g). This latter value is consistent with the 0.1–0.3 µg/g reported for lichens from unpolluted areas [32,33].

**Figure 4:**
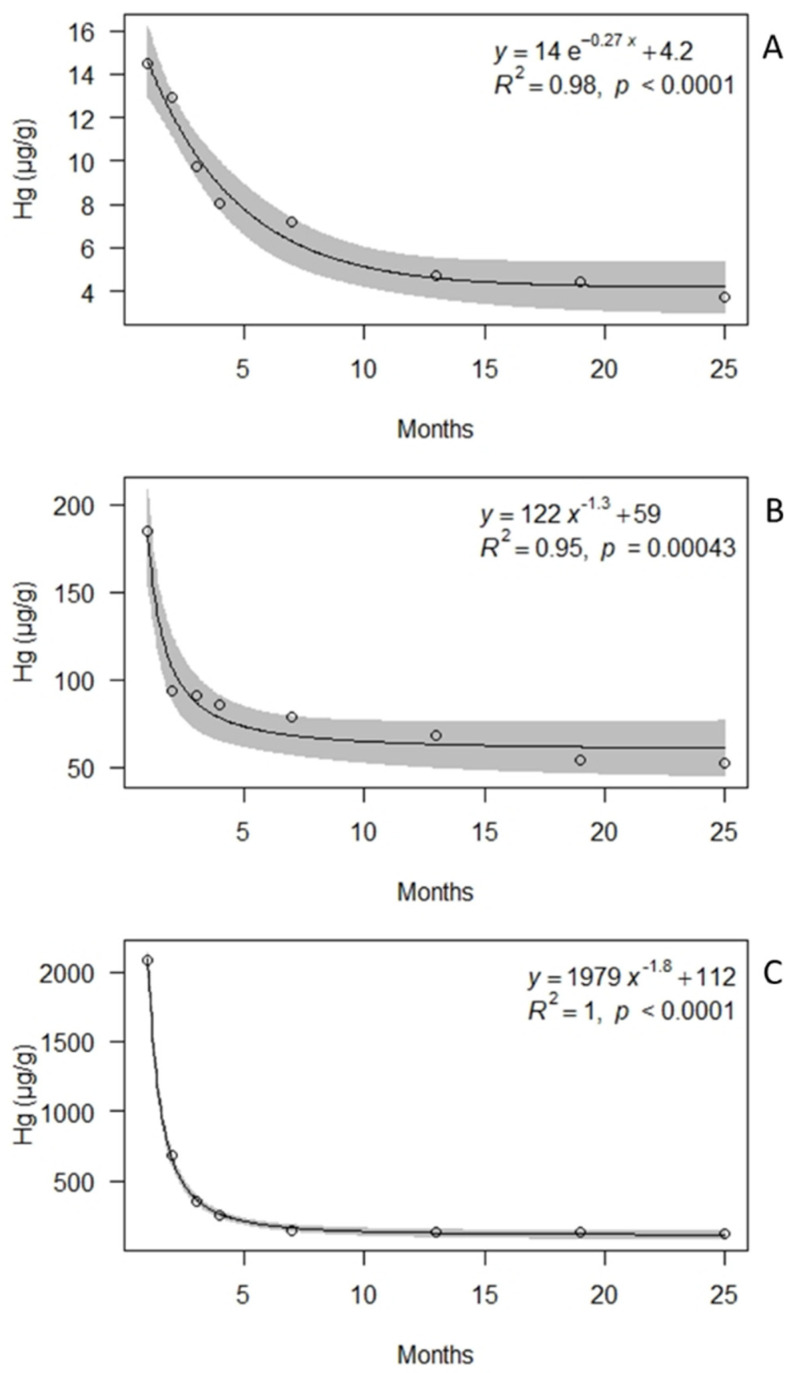
Exponential and power regression of mean Hg concentrations (µg/g) over time in samples of the lichen *Evernia prunastri* incubated with 1 (**A**), 10 (**B**), and 100 (**C**) µM Hg^2+^ solutions and transplanted for 1, 2, 3, 6, 12, 18, and 24 months at the Botanical Garden of the University of Siena. Gray area = 95% confidence interval.

**Figure 5:**
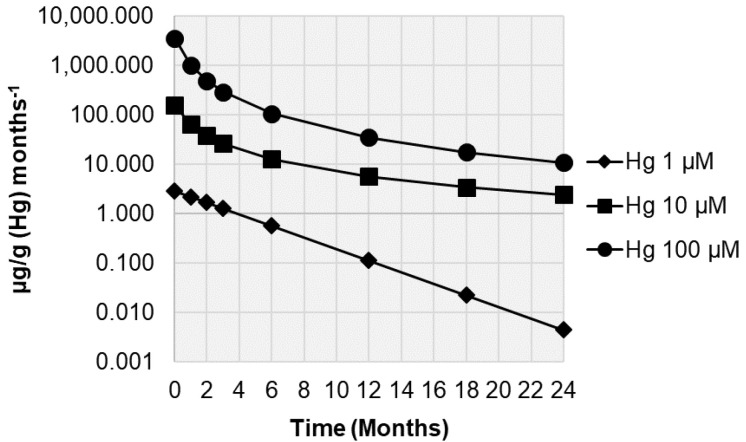
Release rates of Hg (µg/g month^−1^) in samples of the lichen *Evernia prunastri* incubated with 1, 10, and 100 µM Hg^2+^ solutions and transplanted for 1, 2, 3, 6, 12, 18, and 24 months at the Botanical Garden of the University of Siena.

The samples treated with 1 µM Hg showed the best relationship between Hg concentration (µg/g) and time (months) with an exponential regression model, while those treated with 10 and 100 µM Hg showed the best relationship between Hg concentration and time with a power regression model (Figure 4). The calculated release rates (Figure 5) indicate decreases already after one month from the exposure in the Botanical Garden. After 24 months, ongoing release rates were observed for samples treated with 10 µM Hg (ca. 2 µg/g month^−1^) and 100 µM Hg (11 µg/g month^−1^), but were negligible for those treated with 1 µM Hg (ca. 0.004 µg/g month^−1^).

Samples treated with 1 and 10 µM Hg did not show temporal changes (*p* > 0.05) in photosynthetic efficiency and chlorophyll content (Table 1), while those treated with 100 µM Hg showed remarkable physiological damage already after one month (Table 1), with a reduction of ca. 88% and 92%, respectively, when compared with the control value (*p* < 0.05). However, photosynthetic efficiency showed a recovery with time, already after 3 months. After 24 months, the photosynthetic efficiency was ca. 60%. On the other hand, the chlorophyll content recovered insignificantly (*p* > 0.05), at most 10%.

## 4. Discussion

The model species accumulated Hg^2+^ very efficiently and proportionally to the exposure concentration, consistently with the results obtained for the lichens *Cladonia arbuscular* and *Peltigera rufescens* after incubation with 10 to 500 µM Hg^2+^ solutions [14]. After the exposure, samples showed significant reductions in the concentration of Hg across time, with significant decreases being evident already after 1–2 months from the exposure. After 24 months, lichens lost 72–94% of the accumulated Hg, but without reaching control values (mean = 0.23 mg/kg dw). At the end of our experiment, after 24 months of exposure in the Botanical Garden of the University of Siena, an area free from local known sources of Hg, the lichens still retained Hg at concentrations indicating a severe accumulation, according to the bioaccumulation scale proposed by Cecconi et al. [34], i.e., with a bioaccumulation ratio to control values >4.9 (Table 2).

Field studies suggested that lichens may require 2–4 years to return to background concentrations after exposure to atmospheric inputs [25,35,36]. However, the present results showed that, after two years, lichens are only able to reach stable concentrations, but not background ones. As a matter of fact, no significant temporal difference in Hg content was observed as soon as six months after exposure (for samples treated with the highest Hg^2+^ concentration), and 18 months after exposure, all samples showed stable concentrations. This result is supported by the calculated release rates; although, after 24 months, samples still showed ongoing release rates, specifically 2.4 and 11 µg/g month^−1^ for samples exposed to 10 and 100 µM Hg, respectively. These values no longer seem capable of generating further statistically significant reductions from the thalli, being insignificant compared with the respective current Hg concentrations, i.e., 52 and 143 µg/g dw, respectively.

Lichens can reduce their content of heavy metals following several mechanisms (i.e., biomass increase, alternation between hydration and dehydration cycles, competition mechanisms between metals for ion exchange sites, and excretion of metals complexed with oxalates and secondary compounds from the thalli [37,38,39]), among which the reduction of Hg^2+^ to Hg^0^ by solar radiation [40] may be relevant. However, after 24 months, these mechanisms seem to become almost completely undetectable and further reductions seem possible only after several years. Hence, the “residual concentration” of Hg measured after 24 months can thus be considered as the “memory” (sensu [41]) of the simulated past acute pollution episode [42,43], an amount probably associated with the extracellularly bound fraction [44], as only a modest impairment to the photobiont viability was observed. In fact, from a physiological point of view, thalli showed a permanent photosynthetic damage only after exposure to the highest Hg^2+^ concentration (100 µM), thus confirming the results of Pisani et al. [14], which suggested that this metal causes photosynthetic damage at concentrations >50 µM. Strong reductions in the chlorophyll *a* content may be related to the ability of Hg to promote the alteration of the chlorophyll by means of the replacement of Mg in the tetrapyrrole ring, while decreases in the expression of F_V_/F_M_ may be related to the ability of Hg to inhibit the electron transport chain and the activities of the PSII [45,46].

The best approach to background concentrations was observed for samples treated with the lowest Hg concentration (1 µM), thus suggesting that the achievement of a full release may also depend on the initial peak concentration. Similar results were reported by Vannini et al. [27] for lichens exposed to Cu and Zn. In fact, although all treated samples (Cu and Zn 10–100 µM solutions) showed similar amounts of reduction after their exposure in a pristine area (85% release), only samples with a lower initial metal concentration significantly approached unexposed samples. Among the factors involved in reducing metal concentrations in lichens, physiology can also play an important role [47]. Strong physiological impairments or death of the organism may generate the release of ions following damage to the plasmalemma, which in turn may later impair their cytoplasmic immobilization [48]. Consistently, our samples treated with 100 µM showed both an abrupt damage to the photosynthetic system and a very high percentage of release (ca. 94%) after 24 months, albeit a small recovery was observed over time. However, samples that did not show signs of physiological damage (i.e., those treated with 1 and 10 µM Hg^2+^) also showed a high percentage of release (72–74%). As a matter of fact, we may speculate that, in 24 months, the release can reach a maximum of ca. 75% of the accumulated Hg^2+^, suggesting that lichens may release the entire accumulated Hg in less than two years only when its starting concentration (i.e., the concentration immediately preceding the closure of the contamination source) is <1 µg/g. Similar results were obtained for the full release of the accumulated Hg in 2 years by moss [49].

## 5. Conclusions

The laboratory simulation of the exposure of the lichen *Evernia prunastri* to an acute Hg^2+^ contamination event provided useful information regarding the dynamics of bioaccumulation and release of this element over time. Lichen samples accumulated Hg proportionally to the exposure concentration and, after the exposure, reductions over time were evident, already starting from 1–2 months. After 24 months, samples released 72–74 (healthy thalli) to 94% (unhealthy thalli) of the accumulated Hg, but control values of untreated samples were never reached. Depending on the Hg content after the exposure, stable decreased concentrations were reached after 6–24 months. 

The results of this study highlight the ability of the lichen *E. prunastri* to reflect rapidly increasing environmental Hg concentrations, as well as to indicate an ameliorated situation (e.g., the closure of a Hg source). However, we have found evidence that an acute pollution episode can influence the content of Hg in lichens for several years, with the important consequence that the concentration of Hg in lichens does not necessarily reflect its current bioavailability in the study area. As a consequence, caution is necessary when using lichens as a proxy for atmospheric Hg concentrations. Nevertheless, long-term lichen monitoring, especially if supported by atmospheric measurements of time-averaged Hg concentrations (e.g., [50]), is an essential tool for assessing Hg inputs to terrestrial ecosystems.

## Figures and Tables

**Table 1:** Photosynthetic efficiency (F_V_/F_M_) and chlorophyll *a* content (ratio of treated to control values) in samples of the lichen *Evernia prunastri* incubated with 1, 10, and 100 µM Hg^2+^ solutions and transplanted for 1, 2, 3, 6, 12, 18, and 24 months at the Botanical Garden of the University of Siena. Different letters indicate statistically significant (*p* < 0.05) differences between ratios over time.

Time (Months)	Photosynthetic Efficiency (F_V_/F_M_)			Chlorophyll *a* Content		
	1 µM	10 µM	100 µM	1 µM	10 µM	100 µM
0	0.95 ± 0.03 (ab)	0.97 ± 0.02 (ab)	0.13 ± 0.07 (a)	0.99 ± 0.07 (a)	1.01 ± 0.08 (a)	0.09 ± 0.02 (a)
1	1.04 ± 0.01 (a)	0.95 ± 0.03 (ab)	0.23 ± 0.04 (ab)	1.23 ± 0.11 (b)	0.92 ± 0.07 (ab)	0.02 ± 0.01 (b)
2	0.93 ± 0.02 (b)	0.96 ± 0.03 (ab)	0.31 ± 0.03 (ab)	0.93 ± 0.04 (ac)	0.93 ± 0.07 (ab)	0.01 ± 0.01 (b)
3	1.02 ± 0.06 (ab)	1.05 ± 0.02 (ab)	0.35 ± 0.05 (b)	0.97 ± 0.04 (a)	0.92 ± 0.06 (ab)	0.01 ± 0.01 (b)
6	0.98 ± 0.03 (ab)	0.94 ± 0.02 (a)	0.38 ± 0.05 (b)	1.09 ± 0.16 (ab)	0.95 ± 0.9 (ab)	0.01 ± 0.01 (b)
12	0.96 ± 0.01 (ab)	0.96 ± 0.01 (ab)	0.56 ± 0.02 (c)	0.99 ± 0.07 (a)	0.90 ± 0.01 (b)	0.02 ± 0.00 (b)
18	1.00 ± 0.01 (ab)	1.08 ± 0.04 (b)	0.57 ± 0.01 (c)	1.05 ± 0.08 (ab)	1.05 ± 0.06 (a)	0.03 ± 0.01 (b)
24	1.01 ± 0.01 (ab)	0.97 ± 0.03 (ab)	0.63 ± 0.04 (c)	1.08 ± 0.04 (ab)	0.98 ± 0.02 (ab)	0.11 ± 0.02 (a)

**Table 2:** Values of the bioaccumulation ratio to control values after 24 months from exposure.

	T_24_
1 µM	6
10 µM	79
100 µM	230

## Data Availability

The raw data presented in this study are available on request from the corresponding author.
